# Fluorescence Activated Cell Sorting of *Rickettsia prowazekii*-Infected Host Cells Based on Bacterial Burden and Early Detection of Fluorescent Rickettsial Transformants

**DOI:** 10.1371/journal.pone.0152365

**Published:** 2016-03-24

**Authors:** Lonnie O. Driskell, Aimee M. Tucker, Andrew Woodard, Raphael R. Wood, David O. Wood

**Affiliations:** Department of Microbiology and Immunology, College of Medicine, University of South Alabama, Mobile, Alabama, United States of America; Texas A&M Health Science Center, UNITED STATES

## Abstract

*Rickettsia prowazekii*, the causative agent of epidemic typhus, is an obligate intracellular bacterium that replicates only within the cytosol of a eukaryotic host cell. Despite the barriers to genetic manipulation that such a life style creates, rickettsial mutants have been generated by transposon insertion as well as by homologous recombination mechanisms. However, progress is hampered by the length of time required to identify and isolate *R*. *prowazekii* transformants. To reduce the time required and variability associated with propagation and harvesting of rickettsiae for each transformation experiment, characterized frozen stocks were used to generate electrocompetent rickettsiae. Transformation experiments employing these rickettsiae established that fluorescent rickettsial populations could be identified using a fluorescence activated cell sorter within one week following electroporation. Early detection was improved with increasing amounts of transforming DNA. In addition, we demonstrate that heterogeneous populations of rickettsiae-infected cells can be sorted into distinct sub-populations based on the number of rickettsiae per cell. Together our data suggest the combination of fluorescent reporters and cell sorting represent an important technical advance that will facilitate isolation of distinct *R*. *prowazekii* mutants and allow for closer examination of the effects of infection on host cells at various infectious burdens.

## Introduction

*Rickettsia prowazekii* causes the serious and historically significant human disease epidemic typhus. This malady is transmitted by the human body louse and is associated with crowded populations living in unhygienic environments [1–3]. In addition, a zoonotic reservoir, the southeastern flying squirrel, has been associated with sporadic cases of *R*. *prowazekii* infection in the United States as recently as 2009 [4–7]. Due to a low infectious dose and the fact that *R*. *prowazekii* is stable for months in louse feces, there is the potential for aerosol spread and *R*. *prowazekii* was previously weaponized for use as a biological warfare agent [8, 9]. Thus, it is currently classified as a Category B Select Agent.

Rickettsial species are classified into four phylogenetic groups (ancestral, typhus, transitional, spotted fever) with the typhus and spotted fever groups containing some of the most notorious rickettsial pathogens [10, 11]. *R*. *prowazekii* is a member of the typhus group and differs from spotted fever group rickettsiae in several significant ways. *R*. *prowazekii* does not polymerize actin and is unable to spread by this active mechanism from cell to adjacent cell [12, 13]. Also, in contrast to spotted fever group rickettsiae, which induce early damage to the host cell, *R*. *prowazekii* replicates to high rickettsial numbers per cell with little apparent damage until the cell lyses [14–17]. The lack of directional spread to adjacent cells prevents *R*. *prowazekii* from forming distinct, isolatable plaques as proficiently as spotted fever group rickettsiae [18–21]. Similarities in intracellular growth between the different groups are also visible. For example, in cell culture models, rickettsial infections are not uniform and growth within individual host cells, as well as between cells, is non-synchronous. This results in cell populations exhibiting a wide range of rickettsiae per cell. Characterizing the changes in gene expression as a few rickettsiae grow within a cell replete with nutrients to a later stage when there are hundreds of rickettsiae per cell, is hampered by the lack of homogeneous populations of infected cells. Here we describe a protocol to separate cells infected with fluorescent rickettsiae into distinct populations based on bacterial burden.

Despite the challenges an obligate intracellular lifestyle presents to genetic analysis, rickettsial mutants have been generated via transformation using both plasmid and linear DNA [21–28]. Characterization of these mutants has increased our understanding of rickettsial virulence mechanisms[21, 27] and generated an attenuated strain that could serve as a live vaccine based on its ability to grow in culture but not exhibit a virulence phenotype in an animal model [24]. However, in contrast to bacteria that can form colonies on the surface of an agar medium, the identification of *R*. *prowazekii* mutants and the isolation of pure clones is currently a lengthy process. The protocol involves weeks of growth followed by limiting dilution to separate, for example, a transposon insertion mutant from a background composed of other insertions and spontaneously resistant bacteria. As noted above, mutant isolation by the formation of plaques on monolayers, used successfully to purify spotted fever group rickettsial mutants, is also problematic for *R*. *prowazekii*. To circumvent these issues we have taken advantage of the fact that rickettsial species can express fluorescent proteins [23, 28–30]. In combination with antibiotic selection, fluorescent reporters offer a complementary method for the early identification and isolation of rickettsial transformants and for the examination of experimental parameters, such as DNA concentration, on transformant detection. In this report, we describe the utility of this approach in the genetic analysis of *R*. *prowazekii*.

## Materials and Methods

### Bacterial strains, host cell lines, and culture conditions

An *R*. *prowazekii* cloned, transposon insertion mutant, designated Madrid E-RP880::*arr2-Rp/gfp* [23], was used for fluorescence gating experiments. The transposon is inserted into the *R*. *prowazekii* RP880 gene and expresses rifampin resistance (*arr2-Rp*) and the fluorescent protein GFP_UV_. The virulent *R*. *prowazekii* Breinl strain (Passage # 22) was the recipient in the plasmid transformation experiments. Both the Breinl strain and the RP880 mutant were cultured and purified from the yolk sacs of embryonated hen eggs, as described previously [31]. Purified rickettsiae were suspended in a sucrose-phosphate-glutamate-magnesium buffer solution (0.218 M sucrose, 3.76 mM KH_2_PO_4_, 7.1 mM K_2_HPO_4_, 4.9 mM potassium glutamate, and 10 mM MgCl_2_), designated SPGMg, and stored frozen at -80°C. Murine fibroblast L929 cells (American Type Culture Collection, Manassas, VA, ATCC Number CCL-1) were cultured at 34°C with 5% CO_2_ in modified Eagle’s medium (Mediatech, Inc., Herndon, VA), supplemented with 10% heat-inactivated newborn calf serum (HyClone Laboratories, Logan, UT), and 2 mM glutamine (Mediatech, Inc.), designated SMEM. When indicated for the selection of rickettsial mutants, rifampin (Sigma-Aldrich, St. Louis, MO) dissolved in 100% ethanol at 2 mg/ml was added to SMEM to a final concentration of 200 ng/ml. *Escherichia coli* strain XL1-Blue (Stratagene, La Jolla, CA) was used as a recipient for construction and maintenance of shuttle vector pMW1710 and for preparation of plasmid DNA used in rickettsial transformations. XL1-Blue was cultured in Luria-Bertani (LB Lennox) medium at 37°C. For selection of *E*. *coli* transformants, rifampin was added to a final concentration of 50 μg/ml.

### Plasmid construction

A derivative of the rickettsial shuttle vector pRAM18dRGA [32] was generated by replacing the gene encoding GFP_UV_ with a rickettsial codon-adapted gene encoding mCherry (designated RpCherry). This gene was synthesized based on the sequence of mCherry (Clontech, Mountain View, CA) using codons optimized for expression in *R*. *prowazekii* by GenScript (Piscataway, NJ). RpCherry gene expression was placed under the control of the rickettsial *ompA* promoter from *R*. *rickettsii* [28] in a plasmid containing the rickettsial rifampin resistance gene. A cassette (*rpsL*^*P*^*- Rparr-2/ompA*^*P*^*-RpCherry)* was amplified using this intermediate plasmid as template and primer pair DW316/DW1378 (AACATACTTGCTTTTATAGG and GTCGACGGGCCCGGGATCC). The amplified fragment was inserted into pRAM18dRGA digested with BstBI and EagI to remove the GFP cassette (*rpsL*^*P*^*- Rparr-2/ompA*^*P*^*-GFP*_*UV*_) and End-It™ (Epicentre, Madison, WI) repaired to generate blunt ends. The sequence of the resulting plasmid, pMW1710, was verified (Iowa State DNA Facility, Ames, IA) (S1 Sequence). Plasmid pMW1710 was purified using a MP Biomedicals Maxiprep kit (MP Biomedicals, Solon, OH), precipitated with ethanol, and suspended in sterile PCR-quality water at a concentration of ~2.0 μg/μl and stored at 4°C.

### FACS analysis of fluorescent populations of *R*. *prowazekii* infected L929 cells

Flow cytometric analysis of rickettsiae-infected cells was conducted using a Beckman Coulter Moflo XDP contained in a biosafety cabinet located within a dedicated biosafety level 3 (BSL-3) suite. The instrument is equipped with a 100 μm tip. GFP_UV_ was excited using the 488 nm laser and detected using a 529/28 band-pass filter. For sorting, a population of L929 cells infected with an *R*. *prowazekii* transposon insertion mutant, Madrid E-RP880::*arr2-Rp/g*fp [23], that exhibited a range of rickettsiae per cell, was harvested from 175 cm^2^ flasks using trypsin as previously described [25]. Cells were collected by centrifugation at 700 X g, washed once in 10 ml of sorting buffer (1X Dulbecco’s PBS, 1 mM EDTA, 25 mM HEPES [pH 7.0], 1% heat-inactivated newborn calf serum) collected by centrifugation as above and suspended in sorting buffer to a density of ~1 X 10^7^ cells/ml. Cells were placed in a filtered (35 μm) flow cytometry tube (BD Falcon, Franklin Lakes, NJ) for introduction into the MoFlo XDP. Cells from individual flasks were analyzed independently using the same gating parameters and a histogram of cell counts and GFP_UV_ fluorescence was generated.

To generate a population of RpCherry-expressing *R*. *prowazekii* for use in gating experiments, *R*. *prowazekii* Breinl was transformed with 13 μg of pMW1710 and cells infected with fluorescent rickettsiae were sorted on day 13 post electroporation. Prior to analyzing experimental samples, background fluorescence was determined using rickettsiae-infected L929 cells electroporated in the absence of DNA. For experimental samples, the gate was set to exclude any background cells. The sorted cells, positive for RpCherry expression, were then planted into a 25 cm^2^ flask containing 10 ml of SMEM with the following antibiotics: 50 μg/ml gentamicin, 200 ng/ml rifampin, and 1X penicillin-streptomycin-neomycin antibiotic mixture with final concentrations of 50 units, 50, and 100 μg/ml, respectively. Uninfected L929 cells (1x10^6^) were added to provide host cells for expansion of the infection. Medium was changed at 24 hours after sorting, and replaced with SMEM containing 200 ng/ml of rifampin. The 24 hour treatment with the antibiotic cocktail was used to kill extracellular contaminants that may have been acquired during sorting. This treatment did not significantly inhibit rickettsial growth. The infection was amplified and rickettsial growth monitored microscopically by Gimenez staining [33]. Transformed rickettsiae were isolated by ballistic shearing as previously described [22] and the purified rickettsiae stored at -80°C in SPGMg prior to use in infections.

To better represent the heterogeneous populations that result from long-term selection of transformants, two 175 cm^2^ flasks were infected at different (low and high) multiplicities of infection (MOI) with purified pMW1710 rickettsial transformants (see above). After 48 hours of growth, infections were pooled prior to analysis. A sample of each pooled infection was collected prior to sorting to provide a baseline average of the number of rickettsiae per host cell (Presort) compared to the populations collected from two gates representing low and high numbers of rickettsiae per cell. RpCherry was excited with the 561 nm laser and detected using a 625/26 bandpass filter. Three independent infections were analyzed. Parameters for sorting, including selection of gates were identical for each independent infection tested.

### Fluorescence microscopy

For microscopic examination, cells were fixed using 2.5% formaldehyde and cytofuged onto glass slides. Slides were washed with PBS, mounted with Vectashield anti-fade mounting medium (Vector Laboratories, Burlingame, CA), and examined on a Nikon Eclipse TE2000-U microscope using MetaMorph software

### Quantitative PCR (qPCR)

qPCR was used to determine the number of rickettsiae per L929 cell as previously described [26]. Briefly, for rickettsial quantitation, primers DW664/DW665 (CCTGCAAGTAGACATGTGC and AGTGCATTAGCATCAACACC) were used to amplify the *R*. *prowazekii rho* chromosomal gene (RP526). Primers DW1260/DW1261 (CCCTACAGTGCTGTGGGTTT and GACATGCAAGGAGTGCAAGA) were used to amplify the host β-actin gene from the eukaryotic chromosome. To generate template DNA, cells from experimental samples were harvested and processed using the ArchivePure DNA Cell/Tissue kit (5 PRIME, Inc., Gaithersburg, MD). For quantitation analysis, each set of qPCR amplifications was compared to standard curves of either *R*. *prowazekii* genomic or L929 mouse fibroblast DNAs. Assays were performed using the Thermo Scientific Absolute qPCR SYBR Green Mix (Thermo Scientific, Waltham, MA) and a Cepheid Smart Cycler^®^ (Cepheid, Sunnyvale, CA). For GFP_UV_ sorting, rickettsiae and L929 genome equivalents were derived from a single infection sorted three times with each qPCR sample run in duplicate. At least five independent qPCR reactions from three independent infections were used to determine genome equivalents for the RpCherry experiments. Ratios of the number of rickettsiae per L929 cell were calculated from the averages of these replicates. The specificity of amplification was confirmed by melting-curve analysis. Cepheid Smart Cycler Version 2.0c software was used during data acquisition and analysis.

### DNA concentration and the detection of *R*. *prowazekii* transformants

Electroporation of *R*. *prowazekii* was conducted as previously described [22, 25] with one exception. Rather than harvest rickettsiae from infected L929 cell monolayers for each experiment, a large pool of rickettsiae was generated from egg yolk sacs and stored as aliquots at -80°C in SPGMg prior to use. The approximate number of purified, viable rickettsiae in 1 ml aliquots was determined by infecting specific numbers of L929 cells with varying amounts of the pool. The infected cells were planted on cover slips, incubated for 24 hours at 34°C and 5% CO_2_, and visualized by Gimenez staining. The percent of L929 cells infected and the number of rickettsiae per cell were determined microscopically by randomly counting 100 cells and these numbers were used to estimate the number of viable rickettsiae within the aliquots (~3.5 X 10^9^ rickettsiae/ml). To render these bacteria competent for electroporation, rickettsiae were thawed on ice, collected by centrifugation (11,000 x g for 10 minutes at 4°C) and suspended in 20 ml of ice-cold 0.25 M sucrose. Following centrifugation as described above, the supernatant was removed, and the rickettsiae suspended in 400 μl of ice-cold 0.25 M sucrose. A 50 μl aliquot of the competent rickettsiae, along with varied amounts of pMW1710 DNA, was used for each transformation. Rickettsiae were electroporated in 1 mm gap cuvettes using a BTX ECM600 electroporator (BTX Harvard, Holliston, MA) as previously described [22, 25]. Following electroporation, rickettsiae were transferred to a 50 ml conical tube containing 2x10^7^ L929 cells [multiplicity of infection (MOI) ~10–20 rickettsiae/cell] in 3 ml of Hank’s balanced salt solution (Corning, Manassas, VA) supplemented with 5 mM glutamic acid and 0.1% gelatin. Cells were incubated at 34°C at 400 rpm for 1 hour in a Thermomixer R (Eppendorf, Hauppauge, NY). After incubation, rickettsiae-infected L929 cells were planted to three 175 cm^2^ tissue culture flasks containing 25 ml of SMEM and placed in an incubator at 34°C with 5% CO_2_. SMEM medium was changed at 24 hours, and rifampin added to a final concentration of 200 ng/ml for selection of rickettsial transformants. Cells were harvested using trypsin, pooled, and expanded to 6 flasks on day 2 post-transformation and incubated. Fluorescence analysis was performed on days 5–13 as indicated for specific experiments. Medium with rifampin was changed every 2–3 days during the course of the experiment. For analysis of RpCherry-expressing rickettsial transformants, rickettsiae-infected L929 cells were harvested on selected days from one 175 cm^2^ tissue culture flask for each DNA concentration tested. Cells were collected and prepared for analysis as described above. A histogram of forward scatter area (FSC-A) and RpCherry fluorescence area was generated for each DNA concentration tested and the percent of the total population reported.

## Results

### FACS analysis of L929 cells infected with GFP_UV_-expressing fluorescent rickettsiae

**Fig 1:**
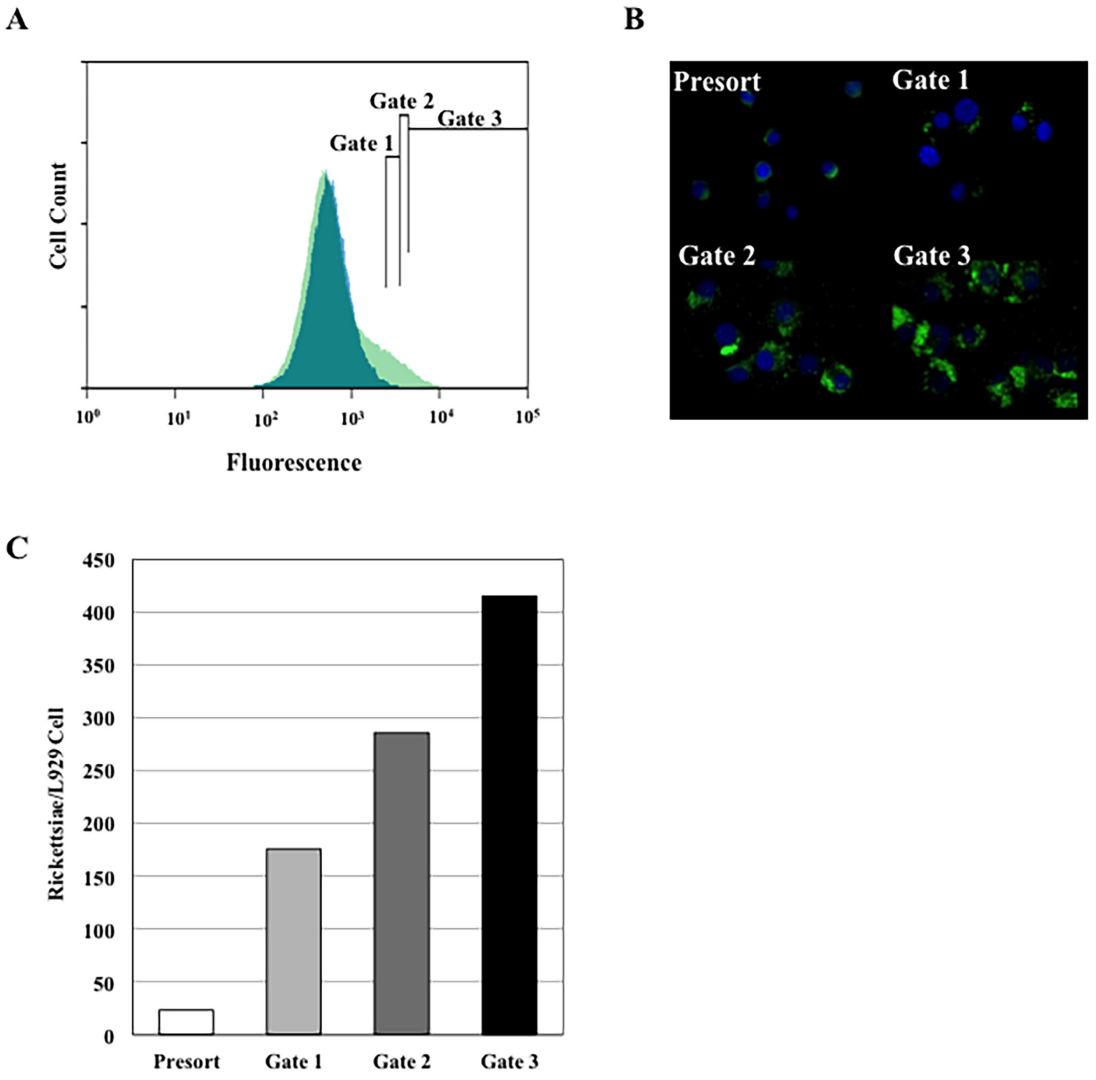
FACS analysis of L929 mouse fibroblasts infected with *R*. *prowazekii* expressing GFP_UV_. A) Fluorescence plot of the cell populations. Uninfected L929 control cells (Blue) and a population of infected cells (Green). Three sorting gates are shown and labeled Gate 1, Gate 2, and Gate 3. B) Fluorescence microscopy of presort and sorted populations. Four panels presenting the presorted cell population, Gate 1, Gate 2; and Gate 3. C) Rickettsiae per host cell for sorted populations. Rickettsial and L929 genome equivalents were derived from a single infection sorted three times with each qPCR sample run in duplicate.

GFP_UV_ is expressed well in several rickettsial species and the gene encoding this fluorescent protein has been used in both transposon and plasmid constructs designed for rickettsial transformations [23, 28]. As its name denotes, optimal excitation of GFP_UV_ would use a frequency in the ultraviolet range. However, in the absence of a UV laser, we examined the general utility of the standard 488 nm blue laser to analyze our existing GFP_UV_ transformant populations. A population of cells resulting from long-term growth of a rickettsial mutant expressing GFP_UV_ (Madrid E-RP880::*arr2-Rp/gfp*) [23] was analyzed and sorted using 488 nm excitation. To test the limits of detection of our systems, we used a culture that, based on staining and light microscopy, contained a high percentage of uninfected L929 cells and a small population infected with a wide range of rickettsiae per cell (Presort). While uninfected L929 cells exhibited considerable auto-fluorescence, cells containing rickettsiae expressing GFP_UV_ could be readily detected above background (Fig 1, Panel A). To establish that fluorescence intensity correlated with increasing rickettsial numbers and to obtain data on gate selection for subsequent experiments, an initial fluorescence profile of uninfected L929 cells was established. Gate 1 was set at the boundary of uninfected L929 cell auto-fluorescence. Subsequent gates, Gate 2 and Gate 3 were set to collect infected cells with increasing fluorescence intensity. The number of rickettsiae per host cell found in each peak was examined both microscopically (Fig 1, Panel B) and by qPCR (Fig 1, Panel C). These data show that host cells containing fluorescent rickettsiae can be separated from uninfected cells and sorted into populations where specific gates harbor infected cells with distinct rickettsial loads.

### FACS analysis and gating of L929 cells infected with RpCherry-expressing fluorescent rickettsiae

**Fig 2:**
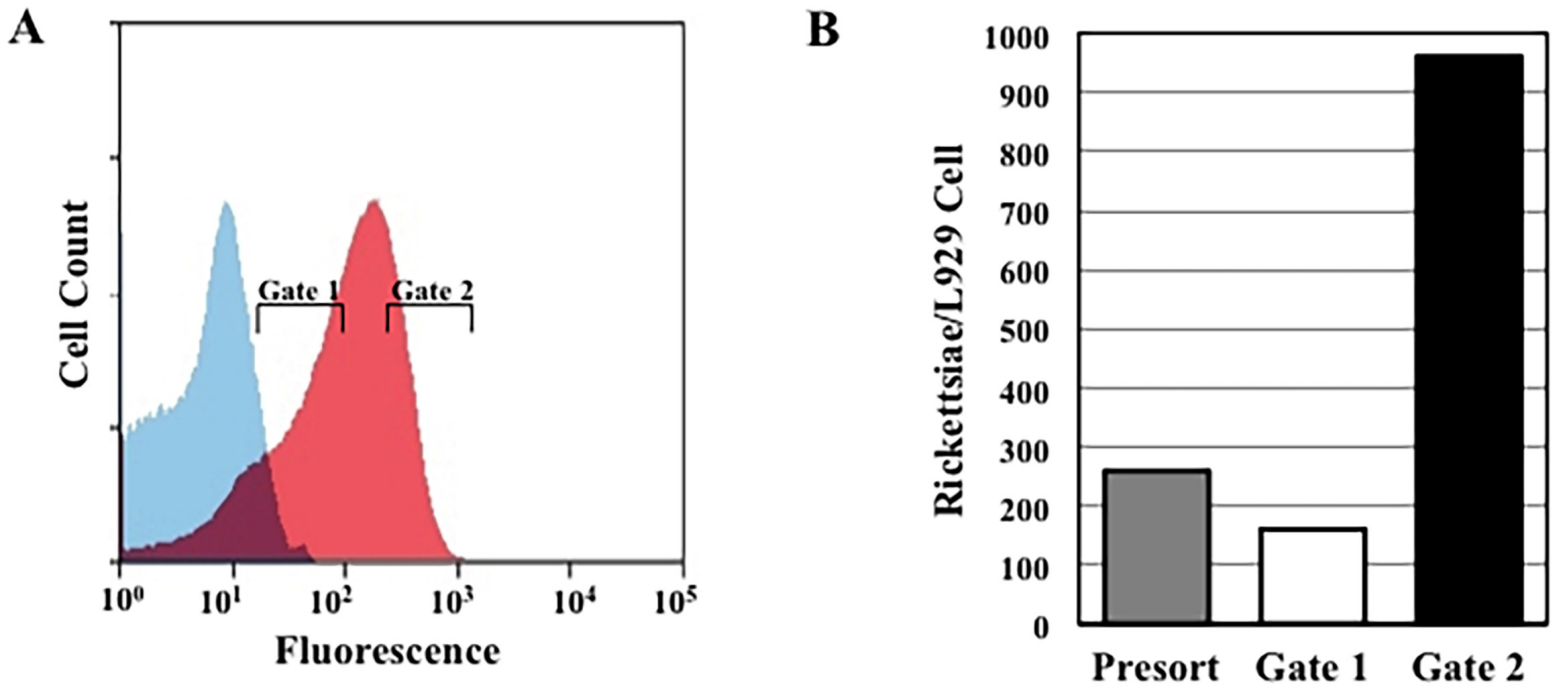
FACS analysis using RpCherry. A) FACS analysis of L929 mouse fibroblasts infected with *R*. *prowazekii* expressing RpCherry. Fluorescence plots of uninfected (Blue) and infected (Red) cell populations are shown. Gates used for sorting lightly infected (Gate 1) and heavily infected (Gate 2) host cells are indicated. B) Data represents the ratio of the average number of genome equivalents of rickettsiae per L929 cells from three independent infections and five independent qPCR reactions for each condition.

To expand the repertoire of fluorescent proteins available for rickettsial studies and to generate a marker that would have a better signal to noise ratio, crucial for identifying rare transformants, we generated a rickettsial codon-adapted mCherry gene, designated RpCherry, to substitute for the GFP_UV_ gene in genetic experiments. In plasmid pMW1710, the RpCherry gene replaces the GFP_UV_ gene of the rickettsial shuttle vector pRAM18dRGA. A population of cells infected with rickettsiae transformed with pMW1710 was analyzed using the 561 nm yellow laser. As seen in Fig 2, Panel A, under these conditions the auto-fluorescence of uninfected L929 cells is low and there is minimal overlap with the fluorescence curve of cells infected with rickettsiae expressing RpCherry. Gating experiments were also performed using this pMW1710 transformed population. As shown by the qPCR results from three independent experiments (Fig 2, Panel B), the RpCherry fluorescent protein can also be used in gating experiments to reproducibly obtain populations of cells with low and high rickettsial loads.

### Effect of DNA concentration on the detection of *R*. *prowazekii* RpCherry-expressing transformants

**Fig 3:**
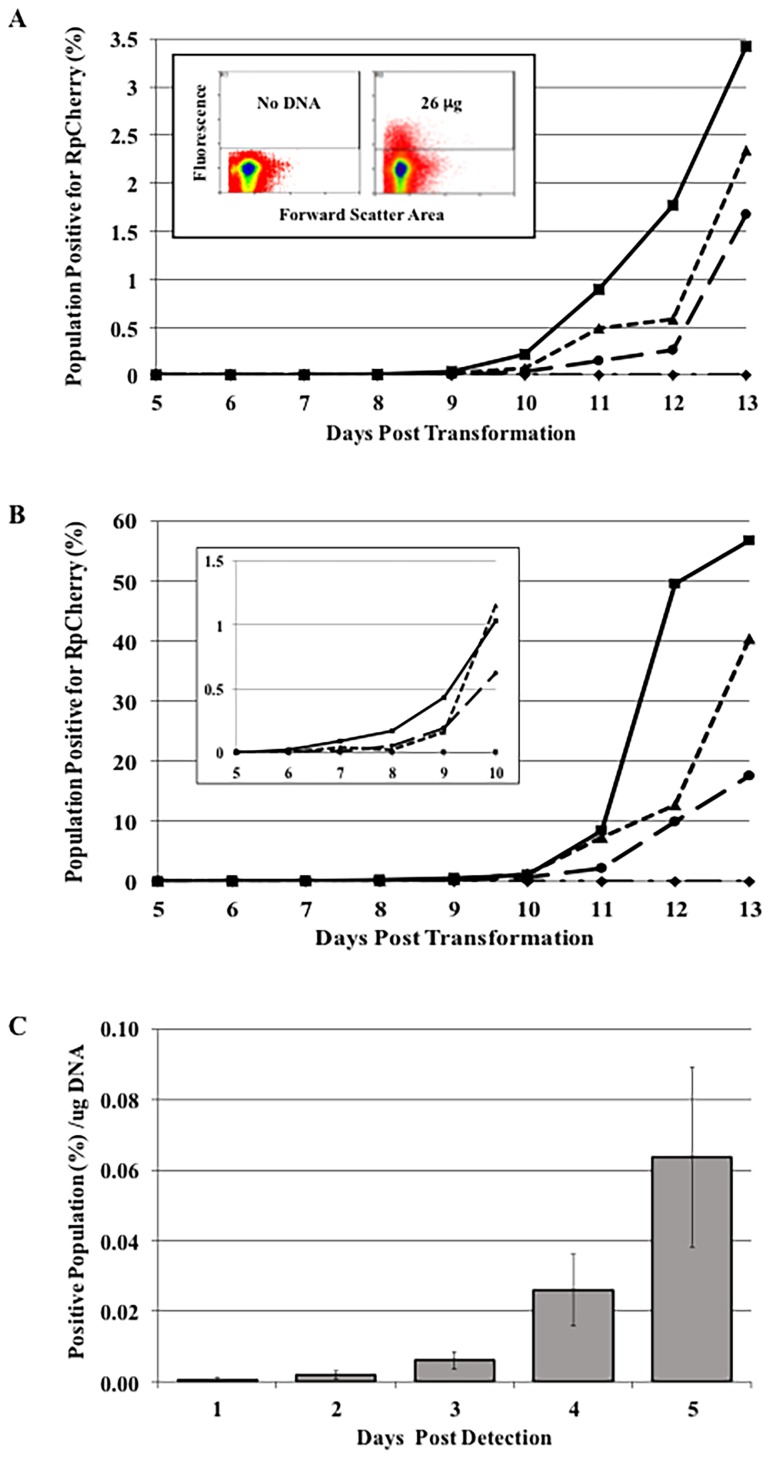
Appearance of pMW1710 transformants based on the concentration of transforming DNA. Competent rickettsiae were electroporated in the presence of increasing amounts of pMW1710 DNA (♦ 0 μg, ● 6 μg, ▲13 μg, ■ 26 μg). Cells were analyzed on days 5–13 and the percent of RpCherry positive cells was determined. Two independent experiments (A and B) were performed. A) Experiment 1; Inset—Analysis was performed at 13 days following electroporation in the presence of 0 μg (Left) or 26 μg (Right) of pMW1710. B) Experiment 2; Inset—Area from days 5–10 on an expanded scale. C) RpCherry positive population per μg of DNA normalized to the first appearance of transformants. Bars represent the average of the two experiments with the range indicated.

The identification and isolation of *R*. *prowazekii* transformants is a lengthy process, involving long-term growth in tissue culture and, if a pure clone is desired, limiting dilution procedures [22, 23]. However, the expression of fluorescent proteins by *R*. *prowazekii* provides a tool for optimizing this process. To demonstrate the potential utility of FACS analysis in rickettsial genetics, we followed the appearance of rickettsiae transformed with plasmid pMW1710. L929 cells infected with control rickettsiae that were mock transformed (no DNA) or transformed with 26 μg of pMW1710 DNA were analyzed 13 days post–electroporation (Fig 3, Panel A, Inset). The no DNA control was used to establish the upper limit of background fluorescence (horizontal line). A fluorescent population above background was readily detected and quantified.

FACS analysis provides a rapid method to quantify and evaluate transformation protocols. One important parameter is the amount of DNA needed to provide the earliest detection and isolation of rickettsial transformants. The effect of plasmid pMW1710 DNA concentration on the appearance of rickettsial transformants is presented in Fig 3, A and B. Two independent experiments are shown in order to demonstrate the variability associated with rickettsial experiments (see y-axis scale differences). Although each of these independent experiments used aliquots from the same egg yolk sac purified rickettsial pool, the manipulations required for rickettsial competence and transformation led to two different levels of initial infection. Experiment 1 (Fig 3, Panel A) exhibited 88–97% infected cells with an average of 7–14 rickettsiae per cell at 24 hours post-infection. In Experiment 2 (Fig 3, Panel B) the initial infection was higher with 96–100% of the cells infected and an average of 23–34 rickettsiae per cell. In the experiment with lower percentages, transformants could be detected by day 8 with the higher concentration of pMW1710 DNA. In contrast, previously published isolations of *R*. *prowazekii* transformants required at least 27 days [22–24, 34]. The number of transformants and the rate of detection increased with DNA concentration, although even the lowest amount of plasmid DNA tested generated transformants. Most importantly, transformants could be detected as early as 6 days following electroporation when using the highest concentration of DNA (Fig 3, Panel B). When normalized to the days after the first appearance of transformants, the percent positive per microgram of DNA for both experiments show similar DNA concentration dependent numbers of transformants (Fig 3, Panel C).

## Discussion

In this paper our goal was to establish fluorescence parameters for sorting *R*. *prowazekii* infected cells into distinct populations based on the number of rickettsiae per cell and to demonstrate the early detection of *R*. *prowazekii* fluorescent transformants. Expression by *R*. *prowazekii* of both GFP_UV_ and a mCherry derivative, RpCherry, were used successfully to isolate, by FACS gating, populations of infected host cells containing distinct bacterial loads. These populations can now be examined for gene expression changes that are hypothesized to occur as *R*. *prowazekii* grows within a cell replete with nutrients to one where the host cell contains hundreds of rickettsiae and is nearing lysis.

FACS gating using GFP_UV_ was accomplished using the standard 488 nm blue laser for excitation. Despite the high background fluorescence exhibited by uninfected cells, we demonstrated that cells containing GFP_UV_–expressing rickettsiae could be differentiated from L929 host cell auto-fluorescence. Thus, stored populations resulting from earlier GFP_UV_ transposon mutagenesis experiments, which are known, from gene rescue experiments, to contain mutants of interest, can now be screened and mutants enriched, expediting cloning. The greater separation between the background fluorescence of uninfected cells and cells infected with RpCherry-expressing rickettsiae will provide an even more efficient tool for mutant identification and isolation.

Refining transformation protocols and accelerating mutant isolation using the FACS were also addressed. Extracellular rickettsiae lose viability over time when isolated from their host cell. Thus, early transformation experiments used rickettsiae isolated from freshly inoculated and propagated infections [25]. In the experiments described here, one major modification to our standard transformation protocol was the use of rickettsiae propagated in hen egg yolk sacs, purified, dispensed into multiple aliquots, and stored frozen at -80°C until needed. This eliminated the additional days needed to repeatedly propagate rickettsiae in L929 cells and to perform a separate rickettsial purification for each transformation. In addition, because yolk sac preparations yield a large quantity of pure rickettsiae, rickettsial viability and infectivity could be determined and the pool used for multiple experiments thereby increasing reproducibility. While the variability inherent in competence induction, electroporation, and infection remains, the use of frozen aliquots removes a significant variable of previous protocols. This also eliminates the two days required to produce competent rickettsiae from L929 cells. This was coupled with the early detection afforded by FACS analysis and the positive effect of increasing transforming DNA concentration. While amounts as low as 1 μg have been used successfully with some rickettsial species [32] our experiments demonstrated that the number of transformants continued to increase from 6 μg up to 26 μg (the highest amount tested). This is in contrast to a previous study that showed no significant increase in *R*. *rickettsii* transposon transformants recovered with increasing DNA concentration (5–20 μg) [35]. This may be due to species differences or transposon versus plasmid transforming DNAs. Most importantly, for our *R*. *prowazekii* experiments, transformants could be detected as early as 6 days after electroporation, a significant improvement over our 23 day standard.

In conclusion, we have confirmed the efficacy of two fluorescent proteins, GFP_UV_ and RpCherry, for identifying and isolating *R*. *prowazekii*- infected cells using laser excitation that is standard for most cell sorters. In addition, we demonstrated that rickettsial populations could be separated based on rickettsiae per host cell, an important advancement for studies examining the effect of rickettsial burden on intracellular growth. In regard to the transformation experiments, since these experiments used plasmid DNA, we have not yet applied these techniques to isolate a pure chromosomal transposon insertion or knockout mutant clone. However the early detection of *R*. *prowazekii* transformants and the ability to sort transformants away from the large population of uninfected cells or those harboring spontaneous resistant mutants will greatly decrease the time, effort and cost associated with the isolation and cloning of a variety of rickettsial mutants by limiting dilution. Eventually, we anticipate the sorting of individual cells containing only one or a few rickettsiae and the propagation of these rickettsiae as cloned populations.

## Supporting Information

S1 Sequence Plasmid pMW1710 Sequence. (DOCX)
