# Supplementary material for: Fluorescence Activated Cell Sorting of Rickettsia prowazekii-Infected Host Cells Based on Bacterial Burden and Early Detection of Fluorescent Rickettsial Transformants
Source: PLoS One. 2016 Mar 24;11(3):e0152365. doi: 10.1371/journal.pone.0152365 (PMC4807063; doi:10.1371/journal.pone.0152365)
Supplement: S1 Sequence — (DOCX) [file pone.0152365.s001.docx]

# pMW1710 Sequence

GGGCGAATTCGAGCTCGGTACCCTCAGCATACATCTTTTTAAGTCGGGCATTCTCCGATTCTAATGCCTTTAGCTTGGTAATCATCGGTAAATCCATACCTCCATACCTTGATCTCTATATAGACATAATTTGGCTGTCACTATGCTTTGATATAGTCATTTTACAGAATCTCCTTTTTTGTTATTAGAAAATTCTACTCTTCTTGTCGATTTTGTTTTAGGGAGGATTACCTTCTCTTATGTTTTTGATCCTGTGTTTTCACTTACTTTGACTATAATTTTTTAACTAATATCCATACTCCACTCTCTTAACCCCTGCACTAGAGCCAAGCCTCTGTATAACATGAGAGTAATTATTACGCACCCAATCCGCAATAAATCTTGCTGGCATTGTAAACTTTAGTGTAGAAGTATCATTGCACTCCTTCACTACTGCTTTTTCAAACCAAGCTGTATACATAGCTTCTCCTAACTCTTCAATTAATCCTTGTCTGATATGATGCCATGCTGTATTTGCTTCGATCGTATCTAATCCCCCACTTTTCGCTAAAATACCTGCATGTGTACCATCAGACACAACATCAGATGTTATAATTTTAGGATATAGCATTACCTTTTCTTCATCTCTCCGCTTTCTTTCATTGCTTGTATCCTCCACCTCTTCTACCTTCTGCACATAATAACCGTTCATTCCATATACTGTTTCTAGCTGCTCACTTAAGATCTCTATTTGTCGCTCACTTAAAGCTAGGTTACTTGGAATTAATACAGTTATAAAGCTGTTGTTGGCACTTATCTTAAACTCTACTTGAGTTAGTATTGAATACGCTATGGAAGTGCTAAATCTTCCTGCTATCTTCTTCTTTACCTGCATTTCTTTACTGGTATCAAATGAATTCTCTATTTGGCTTAAATATTGCTCATGTTCTAACATGTTCTGCTCTTCAGCACCTATATTACAACTGAATCTAAATGTTGTATGATTAACAAGTGGTCCTTGATGTTTTTCGCTCGCAAGCACTTTCTTCATATAGCTTAAGAAGATAAACTTATTCTTAAATTTCTTTTCAGGATATTTGATATATAGCTTCAATAGTAACTGGTTTACAAAGTTAGTGCTAAATTCCCTGTTTGCTTTGAAGTTAAGTATATCTACATCTTTTTCTGATAGTGGATAGAAATGCCTTAATTCTTTATTTTCAGATATATCTTTGATGGTTCTTGCAACAATCTCGGGGTGTATATTAGCACAAGGTAGCTTAAGACTAGCTGTTTCATGGTATTCTTCCTTAGAAACAAAACAACCAGCTTCAGAATCATTTAAAAAAGAAGAATTGTTATAAAATTCAGATGGAAACGAGCTTTGCTCGTTTCCCAAAAACTCTTTTTTGAAATTTTCTTCAGATTTATATATGTTTGAAGAATCATTTATAGATAAATTCTTAAGATTTACATCTATATAATCTATATTTTTAATAATACTATTCTTTGATACAAAGTCTTTACTATATAATTGACCAGAATCAGTGCAAGGAGGGTCATATTCTGTGCATTTCCATTCTTTAGCGATTTCTAAGGCTTTATTGTTATTATACTCAATTACTATTTGTTGCTTTTGCTCTTTCTGTTTTTTCTGATCATTGTTATTTATGAGAGATATATTCAGAAGAGATATTTGCCATAAAGATTTATCAGCCCTGCTATTATAATCATCATTGTTTCTGATAACTAACTTATCTCGCTTAATAAGATTATTATCTTCTAAATCCTTTAATGCTCTAGAAATAGAATTTCTATGTAATTTAAGTTCTTTCTGTAATTCTTGATAAGAAGTGACAATAGAGTGCAGATTATTAGTTATAGAGTGATCATGGTCATAAGCATGATCATAAAAATCTTTGTAATCATTCTTATTGCTGTTACTGGCATCAATATAAGTCTCTGTAGTGATCCTGTTGTCTTTGTCCTTACTGTCCGTGTCTCTGTTACCTTTATCATTATTACTAGATAATGAAGATTTATAAATCTTTGTAAAAAGATAGAGTGTTATAGTCTTTGCTGTTGCAGAAATGCTGCAATTAGAATTTAATTGCTTTAAGAACTCATAAGGTACTCTTATAAACATCTTAGTTTTTTCAAGATGTTGCCTTTTTCCTTCTATGGTTGGCACAAAGATATTATCTATAGAATCTAACTTATCTGACTTAGAGTGACGTGTTTTTGTATTATCGATATCACCTAGATCAAACCTATCAGGTTCATATTTAAGATCATTAAAAACCTTATCAGGTATTGTTGTAGTAATAATATTACGTTTATTCTGACCTTGATCGTTCTTATCTCTAGTAATAATGAAATGTCCTTTAGCTTCCAGGGATTTCTGAAGCACAAATACTTCAGATTTAGAGCAAGATAGTCTTTCTGCCCAGCTTTTAGCAGGTAGAGATATAGATCTATTATGGCCCTTAGAGAAGTTAATACAAGAAAGCCCATCAGCTAAGATGTAGTACATTTTCTCAAGAGTAGATAACTCTTTGCTTGATAATATATGAGATAATACGCACCCAGTTATTGGGACGTATGATTGATTCTTTGTGGTATCATAGTACTGATGTATACCTACATTATCACTGGGCGTAGCAAGCGGGTAAAATGATTGCATTTTATGCTCCTTTTATGGAGCTAAGTACCTGAAACCCTTGACCGCTACGTAGAAATAGTATATATTTATTATATGTAAAAATCATTCTGCCCGTGTTTATTTTTGCATATCATATCAATCAAGGTGTTGAGTTTTAGCTATTTTGAGCACTTAGCACCTGTAGTTTATTGTTTCACTATCGTATCTATTTTATTTAATTTATACTTCTCGAGTTTATCATTTGCAGCATCTATAAACCACTTTCTGCGCGTGATAAAGTTTTCAGCACAAATTTTATCAATCTTTTCTATAATATGCTCTGGAATATCTAAAGATATCCTTTTTACGTTTATTTTCTTCAGCATAAAGCCATTGAGATAAATGAATTATAATCATAGTTAAACATATTTTGTACACATGTCAATAAAAATAATTATTTCACACATAAAAAATAATTATTTTAAAATCAGAGCACATAACATGTATATGTTAACATATATGTATGTTTTTACAATCTAATATAAACATATTGACATATGTATGTTTATATATTTATATTATCGTATAAAAAATAAATAATGTTATTATGATTTTGTCTTTCTTGAATCAAAAAGGAGGTGTAGGTAAGACTACACTTTCAATTAATGTTGCAACATGTCTATCACTAAAAAAGCAAAAAGTGCTTTTAATAGATGCGGATCCACAAAATAGTTCTCTAGATTGGGTTGCAACTCGTAGAAAAGAAAATTTATTCACTGTAGTTGGGCTCACAAAACCAATAATACATAAAGAGGTATCAAAATTGGTTAAGAATTATGATCATATAATTATTGATGGACCACCAAGGATATATGATGTTGCTAAGTCTGCAATAGTTACTAGTGACCTGGTAGTAATGCCAGTGCAACCGTCACCTTATGATATATGGGCAGCTAATGAAGTGGTTAGCCTTATAAAAGAAGTATCTCAACCATTAGAAGGAATAAAAAATATAAAGTCCGCCTTTTTAATTAATAGGAAAATATCCAATACTGCTATTGGTCGAGATGTGGAACAAGCGTTAGATCATTACGGTATGGATATTTTACAAACACATATTTGTCAGAGAGTGGCATATGCAGAAACAGCAGCTATTGGTTCCACAGTTATGGAAAAGCATAATAGAGACAATCTAGCCGTTAGAGAAATAACAGATTTAACAAATGAAATTTTAGAAAAATATAACTAATTGAAAAGGTCATGACAAGAAAAAGAATTGCGCTCCCCAAAAAACCGGTACAAAAAAATACTACTCAATTTATTGATGCATGGGTGTCGGGTAATATAAAACATGAACAACAGGATCAAAAACATAAGAGAACGACTATCTTTTTACCAGAAGAATTGCATAAGAAATTAAAAATCGAGGCGGCTAGGAGGGAGGTAACAATGACTGAAATAATTATAGAAGCTGTAGAAAAAAGTATATAGACATATATATGCTTATATGTCTTAACTTAATGTTTTTGTTTATTAAAGCAAGGATGCTTAATGTTAAGCAGGATCAAATACTATTCATCACGTTTCTTGATCAGACTTATAACTAAATTTTAGTGTAGTACAAATCATATAAACATATAGGAATATGTATGTTTATATTTTGGTCCTACCAATCATTTCATCTATAGCGATTTGAAAATAATCTGCCAGTTTTACTATAACTTTAATACTTAAAGTCCTATTATCACTATTTGTATTAATAAAATGAGTAATGGTACTACTACCTACTCTGCAATTTTTTTCCAATATATACTCTGTAATGTTAAGTTGCTGTATTTTTGTTTTAAGAAAAGAGAATAAGTTATTATTAATATCATTTAAAGTTAAAGATACATATAATTGATATTTTTTTGTATTTTTTATAAATTTTGTGCGACCAAGTACTTCATCTATATCACATTCAAAGAATGTTGCAATTATAAGAATAGTGATGATATTAGGATTAAGTAAAATTGCGTTTAGGAGTTTGCTAATAGTAGATCGCGATAAAGATGTTCCTAATACCAAATCTTTTTGTTGTATTTTATAAGCTTTAATTTTTTGACTTAAAAATTTTTGTATCCTTAGTGCAATATCCATAAATAACTCAAAATATCATACAAATAGTTTCTGAATGCCAAAAACTATTTTCTTGACACATTTCTTGACACACCAAAAATATATTTAACAAAGTAGAAGTATGAGGTAGTGGATTGAGCTGATAATAAAGTACAAACTATCTTATAAAAAGAAAAACCAATAAACACTCAAATTTTGGACAAGGAGCGTGTTGACTGGTGATTAAAGTCACCCAAACTGCAAGATTAGTTCACATATCTTATGACCCTAATGTTACGAGTTTTGTAGATCAAGCGTGCAATGCTGACAATGCAGATCATGATAACCAATCCTCATCAAAATTACCTACCGCTAAAACAATCACAGTGCTAATCAGTTTTTTAAAACTCAGCAAATATGACGATGAGTGGTTAAGAGTAGGGAAGCAACTACTGCTACTGCAAAGACCTACAGATAAGCGAAGATGCTATAGGTCAGTTTTATTATGTTTTAAGTCAGTAATCAAAACCCATTCTGATAATTACGTAGCATATTACTTTAGCGGTATAACATTATGTTTGCTTGGAAGTTATGACGAAGCAATAAAGATGTTTGATATGAGTCTTCGCTATAACAAAGAAAAAAGCACTGCTCTTGTACATACATTTATCTGAGGTATCAAGCATCACAGAGGGCATCAGACTTGCTACAGGAAATAATAACAGTGGTAATAGTGATTGATGCACCCATGCAGAAATGTTTTTTGTTAAATAACGAGAGTTTAACAGTGAATGAGGGTCCTAGACAAGCTAGAGAGGAAAATCATTAAAAAACTTCAAAGATACAAAATAGATGGGGTAGGGAAAATTAGTGCGACTAATCACAAACAACGTTCACCCAAAGGTAAAATATTGGCATCAGCAGAACAAGAAAAATGGGGTAGATATTTTGAACTAGGGATGTATTGGCACGGAAGAGAAGAGGGAAATCATAAAGAATTAATATATTTGAGAAGGTGCTTGCAGCTAAATCCCGAGAATGAACCAGCAAATTGTTATGTGGGGATGATACTGCAAAAGCAAGGCAGATATCATGAGAGCTTAGCGGCTTACAGAAAAGCAATATCATTAAGACCTGAGCTAAAGGAATTAGACTCGAAGAATGCCCGACTTAAAAAGATGTATGCTGAGGGGATCCTCTAGAGTCGACCTGCAGCGGCCAACATACTTGCTTTTATAGGAAAAATAATTATAATTTAAGTTTACTGTAATTTACAAGTTCTTATAATTTGTATTTAGATATGATTTAAAGAGAGTCAAGGGTGAAGTATATTTTTACCCTTTAATCAGTGATATAAGTAAGTTTTTTGGAGTGTTTTCATATGGTAAAAGATTGGATTCCTATTTCTCATGATAATTATAAACAAGTACAAGGACCATTTTATCATGGAACTAAAGCTAATTTAGCAATTGGTGATTTACTAACTACAGGATTTATTTCTCATTTTGAAGATGGTCGAATTCTTAAACATATTTATTTTTCAGCTTTAATGGAACCAGCAGTTTGGGGAGCTGAACTTGCTATGTCACTATCTGGTCTTGAAGGTCGTGGTTATATATATATAGTTGAACCAACAGGACCATTTGAAGATGATCCAAATCTTACAAATAAAAAATTTCCTGGTAATCCAACACAATCTTATAGAACTTGTGAACCTTTAAGAATTGTTGGTGTTGTTGAAGATTGGGAAGGACATCCTGTTGAATTAATAAGAGGAATGTTAGATTCATTAGAAGATTTAAAACGTCGTGGTTTACATGTTATTGAAGATTAAGGATCCTCTAGAGTCGACCTGCAGGCATGCAAGCTTGCCAACGACTACGCACTAGCCAACAAGAGCTTCAGGGTTGAGATGTGTATAAGAGACAGCTGTCTTAATGAATCGGCCAACGCGCGGGGAGAGGCGGTTTGCGTAGGATCCGCATCAAGGCCAACACCGCTACGGAATGACATATAATCTATACATAAAAATATCATCTATGTTATTAATTATAATAGACATATTAAAAAAGTTGCGTTATAACACTTTTTAAGTGATTTAATTTTGATTAAGGTTTTACATATGGTATCAAAAGGTGAAGAAGATAATATGGCTATAATTAAAGAATTTATGAGATTTAAAGTACACATGGAAGGTAGTGTTAATGGTCATGAATTTGAAATAGAAGGTGAAGGTGAAGGTCGTCCTTATGAAGGTACTCAAACAGCTAAATTAAAAGTAACTAAAGGTGGTCCTTTACCATTTGCATGGGATATTTTATCTCCTCAATTTATGTATGGTTCAAAAGCTTATGTTAAACATCCTGCAGATATTCCAGATTATTTAAAATTATCTTTTCCAGAAGGTTTTAAATGGGAAAGAGTAATGAATTTTGAAGATGGTGGTGTAGTTACTGTTACACAAGATAGTTCTTTACAAGATGGTGAATTTATATATAAAGTAAAATTACGTGGTACTAATTTTCCTTCAGATGGTCCAGTTATGCAAAAGAAAACTATGGGTTGGGAAGCTTCAAGTGAAAGAATGTATCCTGAAGATGGAGCATTAAAAGGTGAAATTAAACAACGTTTAAAATTAAAAGATGGTGGTCATTATGATGCTGAAGTAAAAACTACATATAAAGCTAAAAAACCTGTACAATTACCAGGTGCTTATAATGTTAATATAAAATTAGATATTACTAGTCATAATGAAGATTATACAATAGTTGAACAATATGAAAGAGCAGAAGGTCGTCATTCTACAGGTGGTATGGATGAATTATATAAATAGGATCCCGGGCCCGTCGACCGAAAGATCCCAACGAAAAGCGTGACCACATGGTCCTTCTTGAGTTTGTAACTGCTGCTGGGATTACACATGGCATGGATGAGCTCTACAAATAAGGCTAATAAGCAACCGTTCTATAATCATAAAAAAAGCCTATATAGTTTGACTATATAGGCTTTTTTGCTTTATAATGTAGTTTTGAGAAGCGTCATTGCGAGGATCCCCAGCTTGCATGCCTGCAGGCATGCAAGCTTGAGTATTCTATAGTGTCACCTAAATAGCTTGGCGTAATCATGGTCATAGCTGTTTCCTGTGTGAAATTGTTATCCGCTCACAATTCCACACAACATACGAGCCGGAAGCATAAAGTGTAAAGCCTGGGGTGCCTAATGAGTGAGCTAACTCACATTAATTGCGTTGCGCTCACTGCCCGCTTTCCAGTCGGGAAACCTGTCGTGCCAGCTGCATTAATGAATCGGCCAACGCGCGGGGAGAGGCGGTTTGCGTATTGGGCGCTCTTCCGCTTCCTCGCTCACTGACTCGCTGCGCTCGGTCGTTCGGCTGCGGCGAGCGGTATCAGCTCACTCAAAGGCGGTAATACGGTTATCCACAGAATCAGGGGATAACGCAGGAAAGAACATGTGAGCAAAAGGCCAGCAAAAGGCCAGGAACCGTAAAAAGGCCGCGTTGCTGGCGTTTTTCCATAGGCTCCGCCCCCCTGACGAGCATCACAAAAATCGACGCTCAAGTCAGAGGTGGCGAAACCCGACAGGACTATAAAGATACCAGGCGTTTCCCCCTGGAAGCTCCCTCGTGCGCTCTCCTGTTCCGACCCTGCCGCTTACCGGATACCTGTCCGCCTTTCTCCCTTCGGGAAGCGTGGCGCTTTCTCATAGCTCACGCTGTAGGTATCTCAGTTCGGTGTAGGTCGTTCGCTCCAAGCTGGGCTGTGTGCACGAACCCCCCGTTCAGCCCGACCGCTGCGCCTTATCCGGTAACTATCGTCTTGAGTCCAACCCGGTAAGACACGACTTATCGCCACTGGCAGCAGCCACTGGTAACAGGATTAGCAGAGCGAGGTATGTAGGCGGTGCTACAGAGTTCTTGAAGTGGTGGCCTAACTACGGCTACACTAGAAGAACAGTATTTGGTATCTGCGCTCTGCTGAAGCCAGTTACCTTCGGAAAAAGAGTTGGTAGCTCTTGATCCGGCAAACAAACCACCGCTGGTAGCGGTGGTTTTTTTGTTTGCAAGCAGCAGATTACGCGCAGAAAAAAAGGATCTCAAGAAGATCCTTTGATCTTTTCTACGGGGTCTGACGCTCAGTGGAACGAAAACTCACGTTAAGGGATTTTGGTCATGAGATTATCAAAAAGGATCTTCACCTAGATCCTTTTAAATTAAAAATGAAGTTTTAAATCAATCTAAAGTATATATGAGTAAACTTGGTCTGACAGTTACCAATGCTTAATCAGTGAGGCACCTATCTCAGCGATCTGTCTATTTCGTTCATCCATAGTTGCCTGACTCCCCGTCGTGTAGATAACTACGATACGGGAGGGCTTACCATCTGGCCCCAGTGCTGCAATGATACCGCGAGACCCACGCTCACCGGCTCCAGATTTATCAGCAATAAACCAGCCAGCCGGAAGGGCCGAGCGCAGAAGTGGTCCTGCAACTTTATCCGCCTCCATCCAGTCTATTAATTGTTGCCGGGAAGCTAGAGTAAGTAGTTCGCCAGTTAATAGTTTGCGCAACGTTGTTGCCATTGCTACAGGCATCGTGGTGTCACGCTCGTCGTTTGGTATGGCTTCATTCAGCTCCGGTTCCCAACGATCAAGGCGAGTTACATGATCCCCCATGTTGTGCAAAAAAGCGGTTAGCTCCTTCGGTCCTCCGATCGTTGTCAGAAGTAAGTTGGCCGCAGTGTTATCACTCATGGTTATGGCAGCACTGCATAATTCTCTTACTGTCATGCCATCCGTAAGATGCTTTTCTGTGACTGGTGAGTACTCAACCAAGTCATTCTGAGAATAGTGTATGCGGCGACCGAGTTGCTCTTGCCCGGCGTCAATACGGGATAATACCGCGCCACATAGCAGAACTTTAAAAGTGCTCATCATTGGAAAACGTTCTTCGGGGCGAAAACTCTCAAGGATCTTACCGCTGTTGAGATCCAGTTCGATGTAACCCACTCGTGCACCCAACTGATCTTCAGCATCTTTTACTTTCACCAGCGTTTCTGGGTGAGCAAAAACAGGAAGGCAAAATGCCGCAAAAAAGGGAATAAGGGCGACACGGAAATGTTGAATACTCATACTCTTCCTTTTTCAATATTATTGAAGCATTTATCAGGGTTATTGTCTCATGAGCGGATACATATTTGAATGTATTTAGAAAAATAAACAAATAGGGGTTCCGCGCACATTTCCCCGAAAAGTGCCACCTGACGTCTAAGAAACCATTATTATCATGACATTAACCTATAAAAATAGGCGTATCACGAGGCCCTTTCGTCTCGCGCGTTTCGGTGATGACGGTGAAAACCTCTGACACATGCAGCTCCCGGAGACGGTCACAGCTTGTCTGTAAGCGGATGCCGGGAGCAGACAAGCCCGTCAGGGCGCGTCAGCGGGTGTTGGCGGGTGTCGGGGCTGGCTTAACTATGCGGCATCAGAGCAGATTGTACTGAGAGTGCACCTGCGGTGTGAAATACCGCACAGATGCGTAAGGAGAAAATACCGCATCAGGCGCCATTCGCCATTCAGGCTGCGCAACTGTTGGGAAGGGCGATCGGTGCGGGCCTCTTCGCTATTACGCCAGCTGGCGAAAGGGGGATGTGCTGCAAGGCGATTAAGTTGGGTAACGCCAGGGTTTTCCCAGTCACGACGTTGTAAAACGACGGCCAGTGAATTGTAATACGACTCACTATA

**S1 Sequence. Sequence of plasmid pMW1710**
